# An automated skin segmentation of Breasts in Dynamic Contrast-Enhanced Magnetic Resonance Imaging

**DOI:** 10.1038/s41598-018-22941-2

**Published:** 2018-04-18

**Authors:** Chia-Yen Lee, Tzu-Fang Chang, Nai-Yun Chang, Yeun-Chung Chang

**Affiliations:** 1 Department of Electrical Engineering, National United University, Miao-Li, 36063 Taiwan; 2 Department of Medical Imaging, National Taiwan University Hospital and National Taiwan University College of Medicine, National Taiwan University, Taipei, 10002 Taiwan

## Abstract

Dynamic contrast-enhanced magnetic resonance imaging (DCE-MRI) is used to diagnose breast disease. Obtaining anatomical information from DCE-MRI requires the skin be manually removed so that blood vessels and tumors can be clearly observed by physicians and radiologists; this requires considerable manpower and time. We develop an automated skin segmentation algorithm where the surface skin is removed rapidly and correctly. The rough skin area is segmented by the active contour model, and analyzed in segments according to the continuity of the skin thickness for accuracy. Blood vessels and mammary glands are retained, which remedies the defect of removing some blood vessels in active contours. After three-dimensional imaging, the DCE-MRIs without the skin can be used to see internal anatomical information for clinical applications. The research showed the Dice’s coefficients of the 3D reconstructed images using the proposed algorithm and the active contour model for removing skins are 93.2% and 61.4%, respectively. The time performance of segmenting skins automatically is about 165 times faster than manually. The texture information of the tumors position with/without the skin is compared by the paired t-test yielded all p < 0.05, which suggested the proposed algorithm may enhance observability of tumors at the significance level of 0.05.

## Introduction

With rapid modernization and changing food habits, women are being increasingly exposed to carcinogenic factors that lead to various forms of breast cancers. According to statistics regarding causes of deaths released by the Department of Health (Taiwan), the standardized incidence and death rates of female breast cancer patients are 63.2 and 11.6 (per 100,000 persons), respectively; this has been severely threatening the health and lives of Taiwanese women^1^. The annual increase in the number of women with breast cancer is about 3,300 persons in Taiwan, of which about 1,100 persons die annually. The incidence rate of breast cancer among North American women is higher than that for Taiwanese women, but the curative ratio for early detection is higher for North American women. Therefore, screening needs to be increased for early detection and treatment^2^.

**Figure 1:**
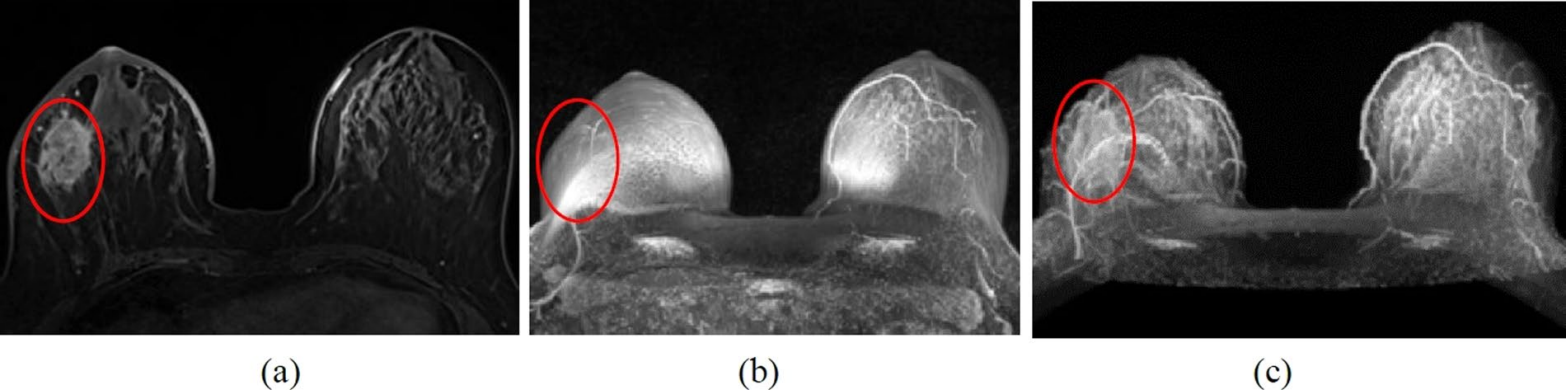
(**a**) Single DCE-MRI image. (**b**) 3D imaging of the original image. (**c**) 3D imaging of skin manually removed from original image. It is very difficult to segment the skin of DCE-MRI because of the following reasons: (1) Each case with 144 DCE-MRI sequence images. (2) There may be blood vessels connected to the mammary glands around the skin in each DCE-MRI (Fig. 2(a)). (3) The skin position and thickness are different in each image (Fig. 2(b)). The aforementioned skin characteristics can be overcome in the skin segmentation algorithm proposed in this study.

At present, the medical imaging procedures used for the clinical monitoring of breast cancer include mammography, breast ultrasound, dynamic contrast-enhanced magnetic resonance imaging (DCE-MRI), positron emission tomography. These procedures have their merits and demerits. It is possible that breast cancers cannot be detected in the early stages because of insufficient spatial resolution^3^; the slight blood vessel proliferation at the very early stages of breast cancer cannot be measured if the screening tools have inadequate sensitivity^4^. It is also possible that screening tools, such as radiation and multiple chemotherapeutic tracking evaluations, are expensive. In clinical tracking, doctors often evaluate the breast cancer treatment effect according to the tumor volume that is reduced in the anatomical image. However, effective treatment may have changed the physiological and biochemical characteristics of the tumor before the changes in the tumor volume are obvious. Currently, DCE-MRI is a high-accuracy clinical checking tool. However, if the DCE-MRI images of the skin are reconstructed into a 3D image, it cannot show the information inside the breast, such as blood vessels and tumors, as the breasts are blocked by the skin. The red circle in Fig. 1(a) shows a tumor. Figure 1(b) shows the DCE-MRI images that are reconstructed into 3D images, and is displayed by maximum intensity projection. Thus, we observe that the same location (inside red circle) cannot see the tumor because of the skin barrier. Figure 1(c) shows the tumor after removing the skin of the breast. Therefore, it is necessary to remove the outer skin to obtain the anatomical information under the skin and to get the anatomical information. It is also possible to segment the tumor by a simple and rapid method in the removed-skin DCE-MRI breast images in the future research. However, Fig. 1(c) was reconstructed by manually removing the skin from 144 DCE-MRI images of each case and was displayed by maximum intensity projection; considerable time and manpower were consumed for this.

**Figure 2:**
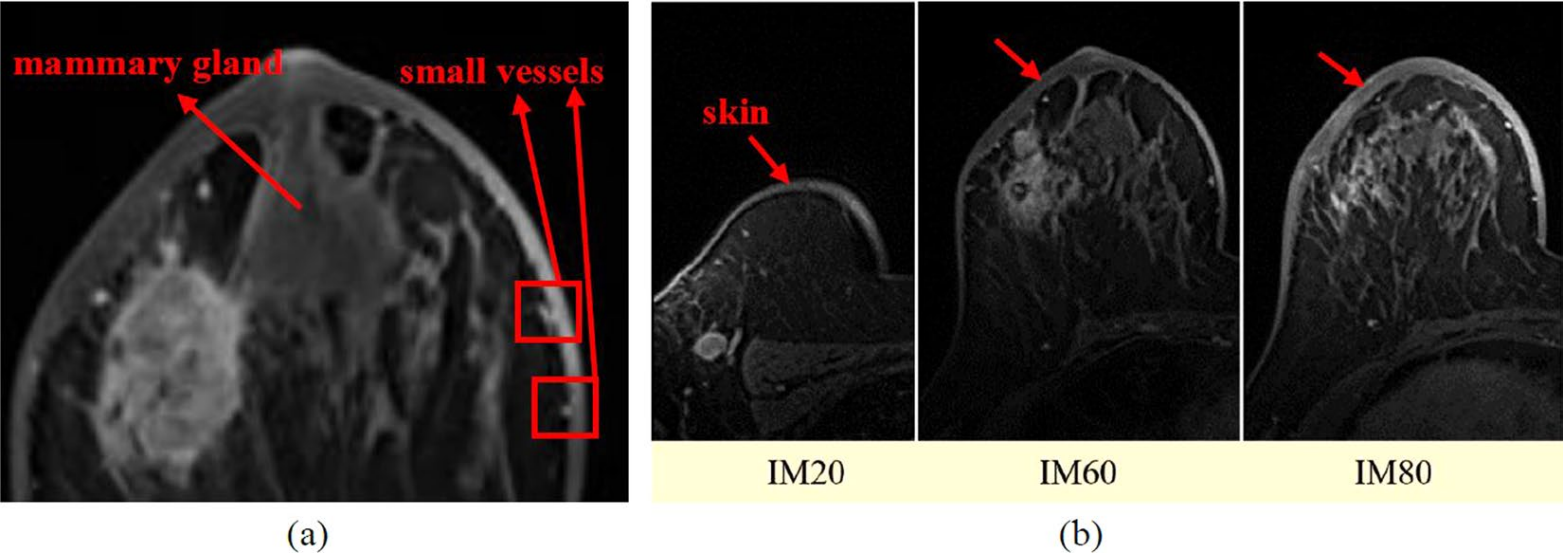
(**a**) Small vessels and mammary glands connected to the skin. (**b**) Skin position and thickness are different in each image.

Skin removal is in the category of image segmentation. In the research area of medical imaging, several famous methods for image segmentation exist, such as the active contour model^5–7^, Level Set Algorithm^8,9^, Seeded Region Growing Algorithm^10^, Watershed^11,12^, and Clustering^13^. The Seeded Region Growing Algorithm gives initial seed points to segments and examines the neighboring pixels to determine if the pixel should be added to the region based on the gray levels. However, this method is not suitable for this study because the small vessels is connected to the skin, and the gray levels are too similar and may be determined as the same region in the red square area of Fig. 2(a). The Watershed and Clustering methods also have the same problem.

Al-Faris *et al*.^14^ suggested the removal of the skin by detecting the skin area using the Level Set Algorithm; then, the thickness of the skin can be minimized using the Thinning Algorithm. However, the demonstration image was too ideal^14^, i.e., the skin thickness was uniform and there was no surrounding noise. One of the main problems that this study overcomes is shown in Fig. 2(b)—the thickness of the skin is dissimilar and the skin position in each DCE-MRI image is unfixed. Moreover, there are many pixels with a similar intensity around the skin; thus, the Level Set Algorithm cannot reach the real skin edge. The skin might have been segmented incompletely or excessively, and small vessels adhering to the skin might have been completely removed. In addition, the processing of the Thinning Algorithm needs to be based on binary images, and an appropriate threshold needs to be selected. However, the skin thickness and noise conditions are different in each DCE-MRI image; therefore, it is difficult to set a threshold applicable to all the 144 DCE-MRI images. At present, the literatures on image segmentation lay emphasis on the segmentation of tumors. Few studies have detected and segmented the skin with non-uniform thickness in the DCE-MRI image. Therefore, a reliable DCE-MRI image skin segmentation algorithm has yet to be proposed.

The active contour model^5–7^ with the internal energy of the cost-function can restrict contour deformation. The external energy looks for pixels nearest to the boundary. This is suitable for this study where the segmented curve shape could be controlled.

Therefore, an automated skin segmentation algorithm is developed that has two parts: detecting the skin area based on the active contour model first and segmenting the skin by skin thickness analysis. Finally, the 3D image could be reconstructed by the stacks of 2D skin-removed DCE-MRI images. The proposed algorithm can assist visual identifying tumors easily and automated delineation of skin boundaries in breast DCE-MRI images

## Results

The images used in this study were provided by the National Taiwan University Hospital (NTUH). Depending on the breast skin characteristics of different patients, the difficulty level associated with removing the breast skin is different; however, the proposed algorithm in this study can be applied to most cases.

### Pre-processing: Removal of internal organs

**Figure 3:**
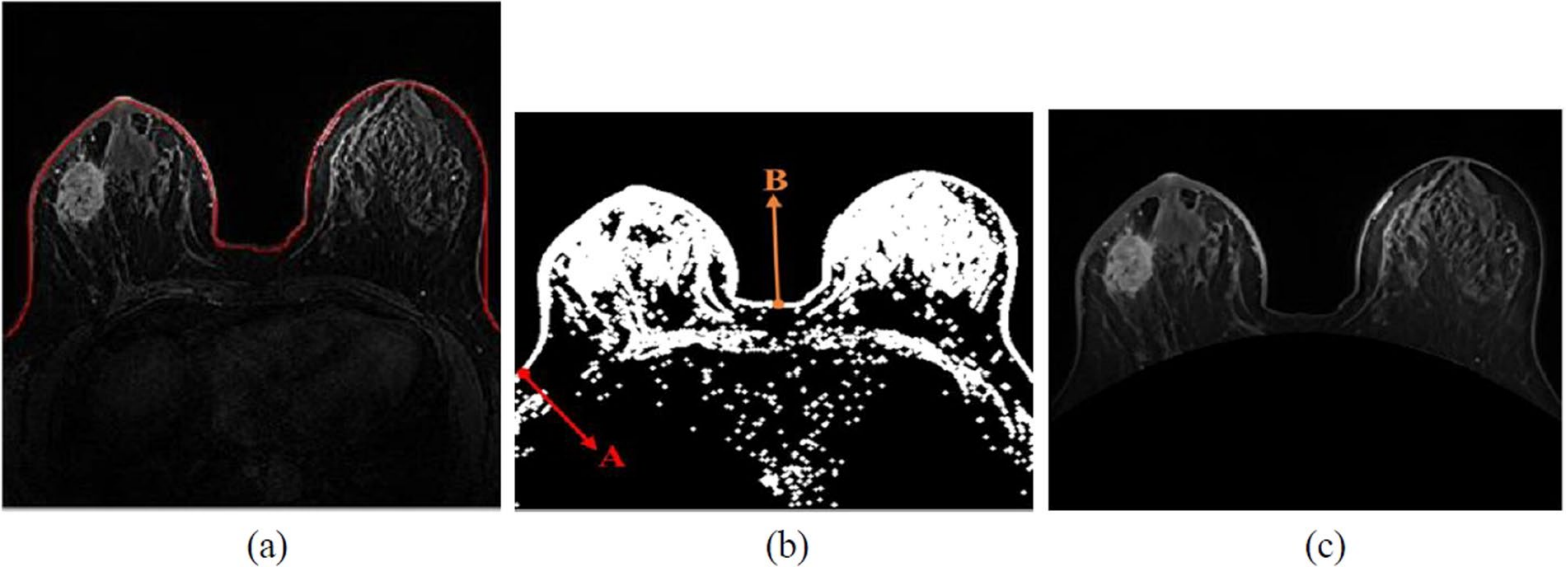
(**a**) Upper boundary of the skin. (**b**) Schematic diagrams of the lowest point A and the highest point B in the binary image and (**c**) the organs-free image.

Noise in the original image was removed by using median filters. Mathematical morphology was used after binarization to enhance the thin area in the skin image. Subsequently, the breast image with complete upper boundary was obtained (Fig. 3(a)); this image can be used to further remove the internal organs. Besides, the upper boundary could be the initial contour for the breast skin detection step in this study. According to the positions of the breast and internal organs, the lowest point A and the highest point B are located at the top and bottom in the left and the middle of the image, respectively (Fig. 3(b)). The parabolic equation was calculated using (2). After curve segmentation, an organs-free image was obtained (Fig. 3(c)).

### Breast skin detection

**Figure 4:**
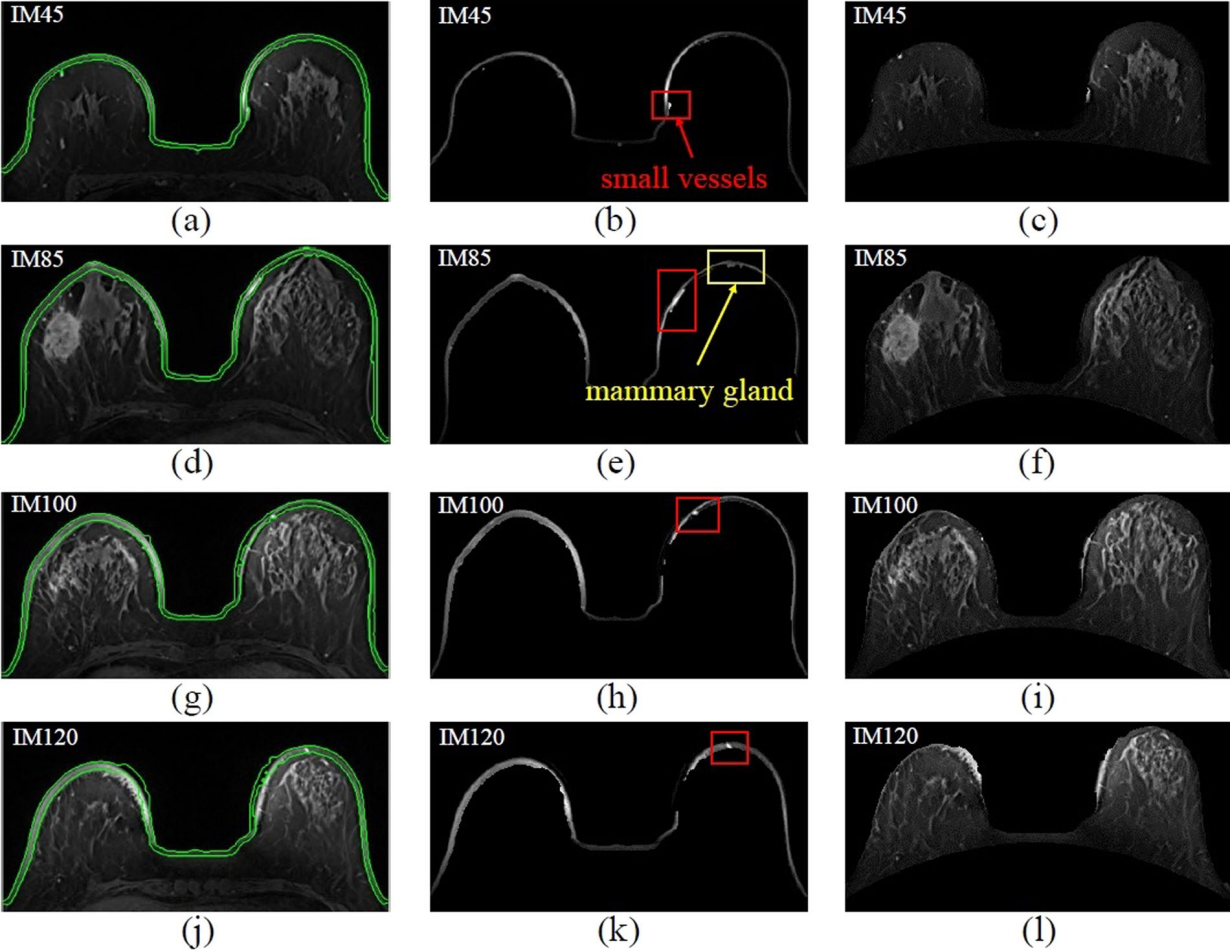
Active contour model algorithm for the breast skin image. Column 1:(**a**)(**d**)(**g**)(**j**) Segmentation result of active contour. Column 2: (**b**)(**e**)(**h**)(**k**) Segmented skin. Column 3: (**c**)(**f**)(**i**)(**l**) MRI images without the skin and internal organs.

The upper boundary of the skin was used as the initial contour of this step. The active contour model was applied to segment the breast skin. The deformation results are shown in Fig. 4. The segmented skins are shown in Fig. 4(b,e,h and k). Small vessels and mammary glands connected to the skin surface were segmented together in this step. Therefore, in the following part of this study, the skin thickness will be analyzed to increase the accuracy of the skin segmentation result.

### Breast skin thickness analysis

**Figure 5:**
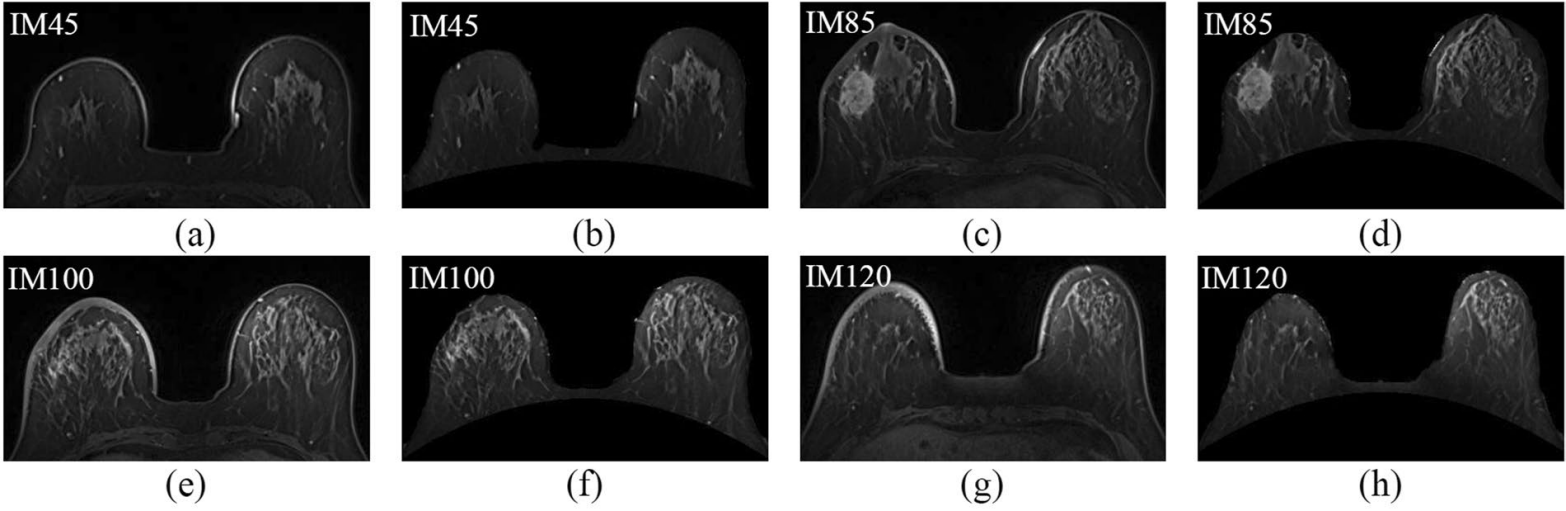
(**a**), (**c**), (**e**), and (**g**) are the original images. (**b**), (**d**), (**f**), and (**h**) are the breast skin segmentation results obtained from the proposed algorithm.

The skin has been rough segmented by the active contour model before thickness detection and analysis. The extracted upper boundary of the skin is numbered forward, and the normal vector in the number sequence direction is analyzed. The skin location was defined by boundary detection. The skin thickness, i.e., the distance between the upper and lower boundaries of each segment of the skin was recorded. The skin thickness of each strip segment was recorded and corrected according to the continuity of the skin thickness. The corresponding skin thickness was segmented as shown in Fig. 5(b,d,f and h). Small blood vessels and mammary glands were retained. Moreover, the breast skin segmentation results obtained from the proposed algorithm were more accurate than that obtained from the active contour model.

## Discussion

**Figure 6:**
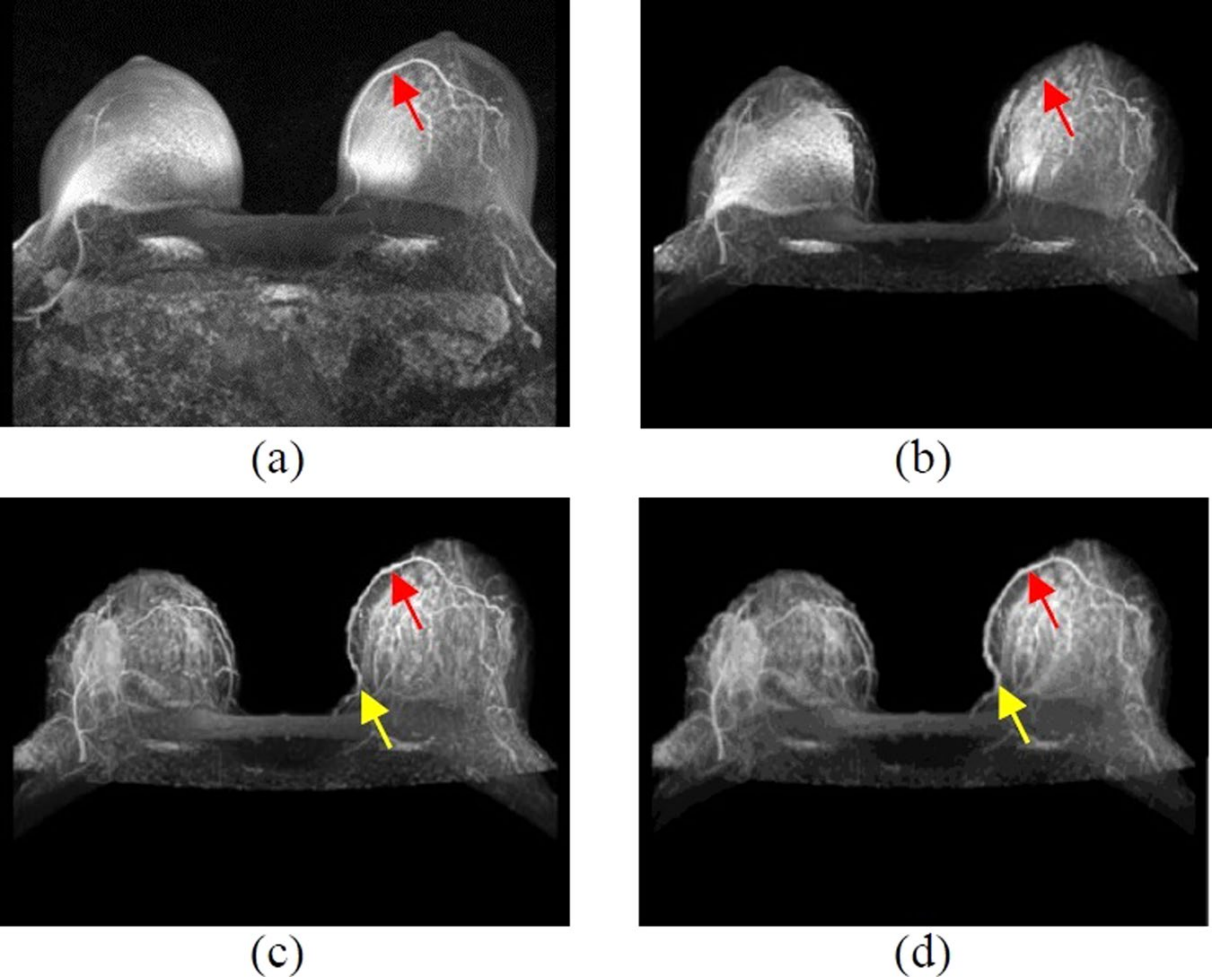
3D imaging displayed by maximum intensity projection. (**a**) Original image. (**b**) Removing the skin by the active contour model. (**c**) Removing the skin by the proposed algorithm. (**d**) Removing the skin manually removed from the original image.

The skin-segmented 144 breast DCE-MRI images are fused into 3D images and displayed at maximum projection. A comparison between the images with and without skin and internal organs shows that the tumor can be easily observed compared with the original image, and the peripheral blood vessels are clear (Fig. 6). It is observed that if the active contour model is used only to segment the skin, the segmentation precision is not good in Fig. 6(b). Therefore, the model should be combined with the proposed skin thickness analysis to have a better effect (Fig. 6(c)). A manually segmented breast skin is shown in Fig. 6(d). It seems no significant different between Fig. 6(c) and Fig. 6(d) in visualization, that’s because these images are presented by maximum intensity projection (MIP), which consists of projecting the voxel with the highest attenuation value on every view throughout the volume on a 2D image. Therefore, the different in detail is not observed easily in the MIP image. The blood vessels indicated by the yellow arrows in Fig. 6 (c) and Fig. 6(d) have slight differences. Compared with Fig. 6 (d), the blood vessel obtained by the proposed algorithm is finer. The reason is the intensity of vessels is similar to that of the skin, so that the blood vessels buried in the skin layer. This causes few blood vessels to be removed when segmenting the skin, but this study still retains most of the blood vessels.

To evaluate if the proposed algorithm can assist to identify the tumor easily, we used a statistical method, gray-level co-occurrence matrix (GLCM), to examine texture properties the tumor^15^. The variables of texture features include Angular Second Moment (ASM), entropy, contrast and correlation^16^. We can speculate that if removing the skin, the tumor location can be found and further to calculate its texture information. If the skin blocks the tumor, then the texture information in the same position will be different. In this assessment, the mean ASM, entropy, contrast and correlation of the tumor before and after removing the skin was illustrated in Table 1. Angular Second Moment is a measure of homogeneity of an image, which means ASM is high when pixels are very similar. Entropy measures the disorder of the image. A non-uniformity of the grayscale distribution or texture crudeness image was associated with a high entropy. Contrast represents local intensity variation or the depth of texture grooves. Deeper texture grooves have a high contrast and better visual sharpness. Correlation means the consistency of image texture.

**Table 1:** Comparison of different texture features of tumors with/without the skin.

Texture	With skin	Without skin	p-value
*ASM*	0.248 ± 0.149	0.168 ± 0.092	0.047
*Entropy*	1.918 ± 0.509	2.315 ± 0.593	0.003
*Contrast*	0.273 ± 0.078	0.429 ± 0.145	0.011
*Correlation*	1.277 ± 0.939	0.806 ± 0.636	0.017

The entropy and contrast of tumors without the skin have been demonstrated to be higher in comparison to those of tumors with the skin, while the ASM and correlation of tumors without the skin are lower than those of tumors with the skin, which clearly showed that the proposed algorithm can see the tumor easily due to remove the skin. The paired t-test on these two sets of mean ASM, entropy, contrast and correlation yielded p-values of 0.047, 0.0034, 0.011, 0.017 (all p < 0.05), respectively, which confirmed that the proposed algorithm may enhance the observability of the tumor at the significance level of 0.05.

While the first assessment concluded that the visual quality of the lesions can be augmented by the proposed skin segmentation algorithm. The second assessment was to further justify that performance of the proposed algorithm. We used Dice’s coefficient to measure for volume similarity. To evaluate the effectiveness of the proposed algorithm, two radiologists with 18- and 30- year experience in breast DCE-MRI delineated the surface skin of different slices as the ground truth. The Dice’s coefficient of the 3D reconstructed image by the proposed algorithm automatically is 93.2%. The Dice’s coefficient of the 3D reconstructed image the active contour model algorithm automatically is 61.4%. In the third assessment, we calculate the time performance of segmenting skins automatically and manually. Physicians and radiologists segment the skin manually, which takes an average of 5 hours for each case (144 images). Instead of removing the skin automatically by the proposed algorithm, it takes only 108 secs for each case (144 images).

## Methods

This study was approved by the Institutional Review Boards of the National Taiwan University Hospital (NTUH). All the experimental methods were performed in accordance with the approved guidelines. Written informed consent was obtained from all patients involved in this study.

**Figure 7:**
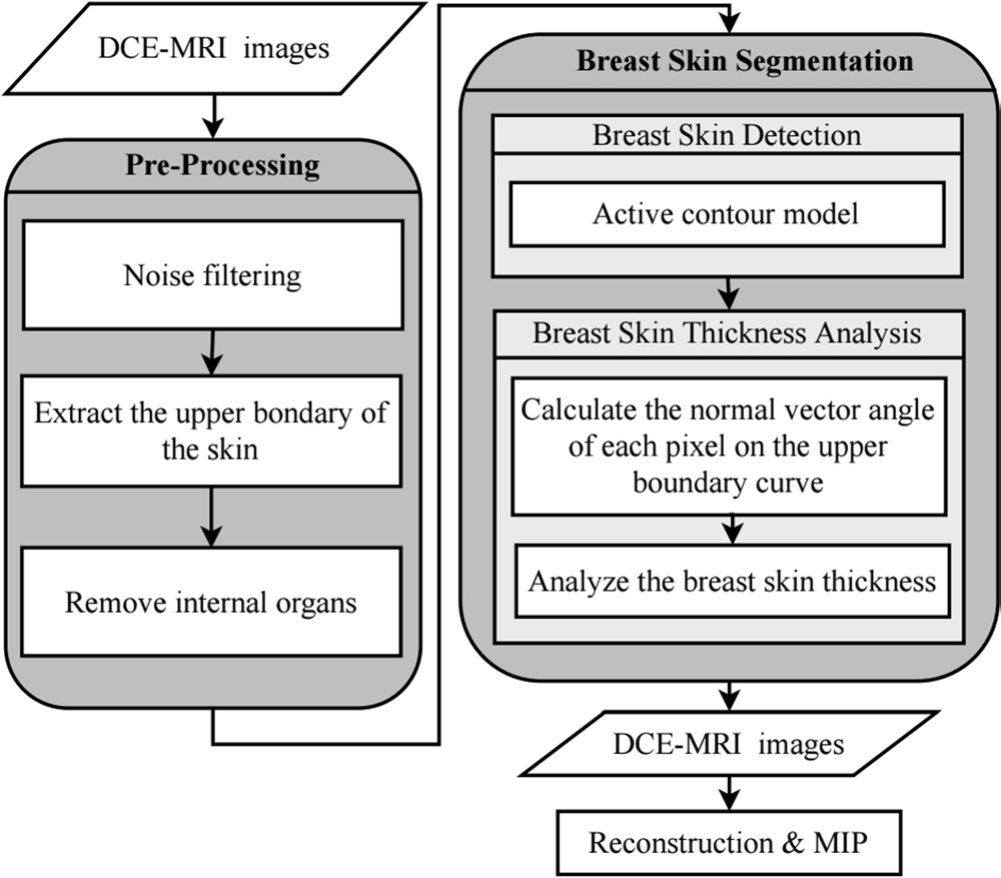
The flowchart of the proposed algorithm.

The flowchart of the proposed algorithm is shown in Fig. 7. The pre-processing stage uses a median filter to remove noise and a mathematical morphology to correct the thin or broken area of the skin. To avoid the internal organs disturbing the detailed breast tissue information in 3D imaging, the organs must be removed in this step. Afterwards, the active contour model is applied to the breast skin detection part to detect the breast skin. The detected skin may be connected to small vessels and mammary glands. Therefore, we developed the skin thickness analysis algorithm. To determine the continuity of the thickness of the skin and to achieve skin segmentation, this algorithm is used as a reference to avoid segmentation into the blood vessels and mammary glands. Finally, the 2D skin-removed DCE-MRI sequence image constructs the 3D image, which can be displayed by maximum intensity projection. The limitation of the proposed algorithm is that the subject undergoes a mastectomy.

### Pre-processing: Removal of internal organs

The median filter is used to remove the salt and pepper-like tiny speckles from the image. The opening and closing of the mathematical morphology are used to correct the thin or broken areas of the skin so that the upper boundary of the skin can be found in the beginning. The internal organs are removed according to the positions of the internal organs and breast tissue. The segmentation curve can be modeled as the parabola opens downward in the shape of an inverted U with a negative constant c.

Let the highest point coordinate be the parabola vertex (${{\rm{x}}}_{{\rm{h}}},{{\rm{y}}}_{{\rm{h}}}$) as the point B of Fig. 3, the parabolic equation as following:

1 $${\rm{y}}=-\,c{({\rm{x}}-{{\rm{x}}}_{{\rm{h}}})}^{2}+{{\rm{y}}}_{{\rm{h}}}$$

### Breast skin detection

The active contour model is used to extract the contour features of the 2D image, and it produces a curve close to the contours of the object. The generated curve is modeled as a snake; therefore, it is also known as Snake. Kass *et al*. first proposed a mathematical model based on the feature of energy variation^5^. Snake is the function of energy minimization. The internal force ${E}_{int}$ restricts the shape, and the external force ${E}_{ext}$ guides the curve behavior. The position of the snake is represented by a parametric curve ${\rm{v}}({\rm{s}})=({\rm{x}}({\rm{s}}),\,{\rm{y}}({\rm{s}}))$, the energy function can be written as equation (2).

2 $${E}_{snake}\,={\int }_{0}^{1}{E}_{int}(v(s))+{E}_{ext}(v(s))ds$$

3 $$={\int }_{0}^{1}\frac{1}{2}(\alpha {|v^{\prime} (s)|}^{2}+\beta {|v^{\prime\prime} (s)|}^{2})+{E}_{ext}(v(s))ds$$

The internal force ${E}_{int}\,\,$as in equation (2) can be divided into the continuity and smoothness of the contour. The coefficients α and β of equation (3) are user-defined, which control the sensitivity of the stretch and the curvature of Snake, respectively. The external force ${E}_{ext}\,\,$consists of the image energy $\,{E}_{image}$ and the constraint energy ${E}_{Constraint}$, which are expressed as following:

4 $${E}_{ext}={E}_{image}+{E}_{Constraint}\,$$

where the image energy ${E}_{image}$ is the energy of the contour curve may be attracted to the salient features of the target object, such as lines, edges or terminations. ${E}_{image}$ can be composed of ${E}_{line}$, ${E}_{edge}$, ${E}_{term}$. The total image energy can be expressed as a weighted combination of the three energy functions, which can be represented as equation (5).

5 $${E}_{image}={w}_{line}{E}_{line}+{w}_{edge}{E}_{edge}+{w}_{term}{E}_{term}$$

where $w$ is a weighting function. ${E}_{line}$ is the intensity of the image as $\,I(x,y)$. ${E}_{edge}$ is the image gradient $\,\mathrm{as}-{|\nabla I(x,y)|}^{2}$. ${E}_{term}$ is the curvature of level lines in a slightly smoothed image, can be written as equation (6)^5^.

6 $${E}_{term}=\frac{{C}_{yy}{C}_{x}^{2}-2{C}_{xy}{C}_{x}{C}_{y}+{C}_{xx}{C}_{y}^{2}}{{({C}_{x}^{2}+{C}_{y}^{2})}^{\frac{3}{2}}}$$

The constraint energy ${E}_{Constraint}$ is the energy that controls and constrains the model curve according to the image information of the real contour. It can be used to interactively guide the Snake toward or away from salient features.

In this study, the active contour model is used to detect a part of the breast skin. The upper edge of the breast skin is taken as the initial contour of the active contour model. After the iterative process, the breast skin can be segmented. According to the results of the segmentation, small blood vessels and mammary glands connected to the skin are divided into objects, and the result would be the preliminary segmentation for breast skin thickness analysis.

### Breast skin thickness analysis

**Figure 8:**
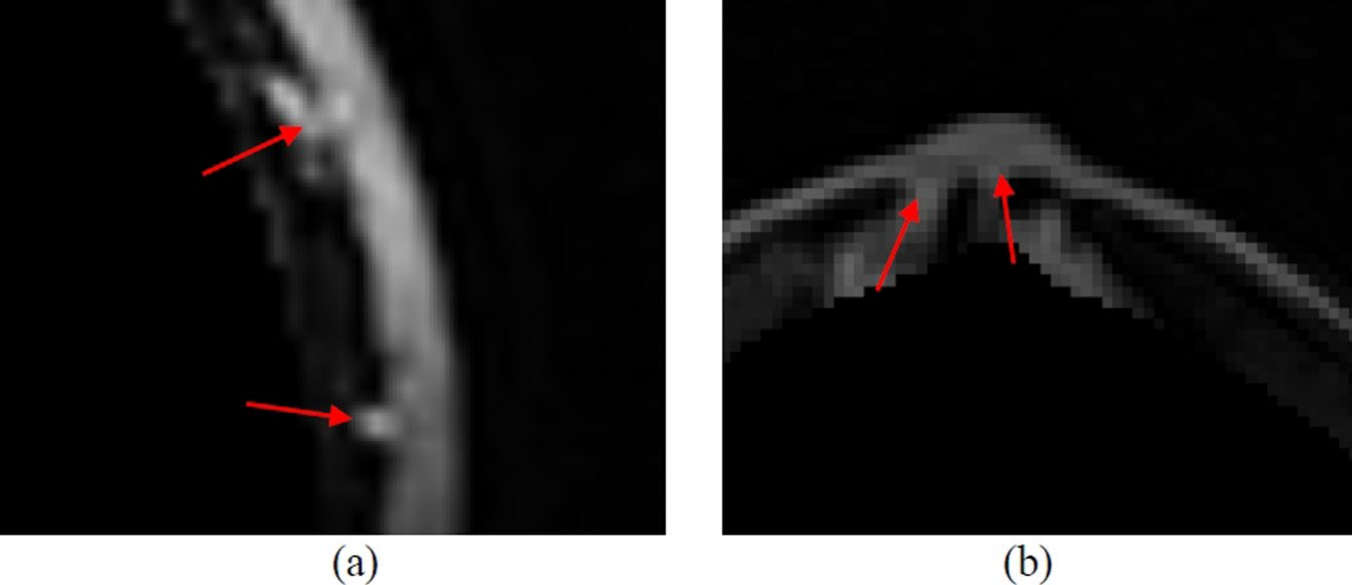
Result of skin detection on (**a**) small vessels connected to the skin and (**b**) mammary glands connected to the skin.

**Figure 9:**
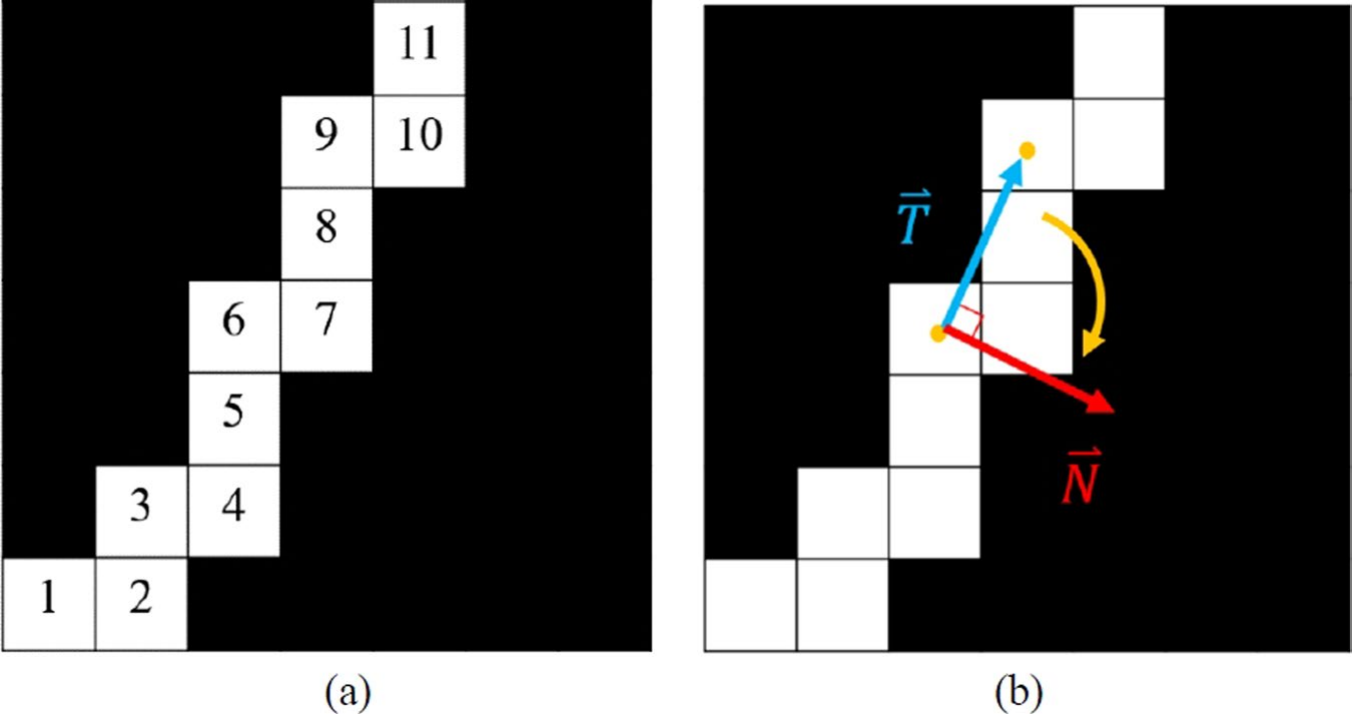
Schematic diagram of the forward numbers of the upper boundary curve. (**b**) Schematic diagram to calculate the normal vector.

The skin thickness analysis algorithm proposed in this study is a solution to the problem where the active contour cannot distinguish blood vessels and mammary glands connected to the skin. As the skin is connected to blood vessels and mammary glands, the skin segmented by the active contour is thicker, shown by the arrows in Fig. 8. The preliminarily segmented skin is non-uniform, which means that this segment not only contained skin but probably also contained blood vessels and mammary glands connected to the skin. The changes in thickness of the normal skin were continuous with thickening and thinning. Therefore, in this study, we successfully analyzed breast skin thickness for removing connective tissue. The normal vector was calculated by taking the first pixel of each column on the upper boundary corresponding to the first pixel of the next columns. Then, the tangent direction $\,\mathop{{\rm{T}}}\limits^{\rightharpoonup }$ was calculated, and it was rotated 90° in the clockwise direction to obtain the normal vector of the upper boundary $\mathop{{\rm{N}}}\limits^{\rightharpoonup }$ (Fig. 9). This vector was used for thickness analysis and skin segmentation of the preliminarily segmented skin.

7 $$\mathop{{\rm{N}}}\limits^{\rightharpoonup }=\frac{\mathop{T\text{'}}\limits^{\rightharpoonup }({\rm{s}})}{\mathop{T\text{'}}\limits^{\rightharpoonup }({\rm{s}})}$$

## Conclusion

To ensure that the skin does not block the anatomical information of tissues in the breast after 3D imaging, removal of surface skin is important. This study uses the active contour model and the skin thickness analysis algorithm in the skin segmentation of breast DCE-MRI image. The proposed method can segment the skin automatically. The tumor location, size, or vascularity can be observed directly in the 3D imaging of breast DCE-MRI, and these anatomical data can assist physicians and radiologists.

Three assessments had been performed to justify the effectiveness of the proposed algorithm. The first assessment conclude that the observability of the tumor could be effectively improved by the proposed algorithm. Quantitatively, the second assessment corroborated that the segmentation performance of the proposed algorithm is better than traditional segmentation algorithm. The third assessment further showed the time performance of the proposed algorithm is faster than segmenting skins manually.

Breast DCE-MRI can play as an adjunct diagnostic tool to ultrasound and mammogram, also can play as a monitoring tool for evaluating chemotherapy treatment of breast cancers. The proposed skin segmentation on the breast DCE-MRI image system can provide diagnosticians with more accurate anatomical information and faster than manually.
